# Advanced Micro- and Nano-Gas Sensor Technology: A Review

**DOI:** 10.3390/s19061285

**Published:** 2019-03-14

**Authors:** Haleh Nazemi, Aashish Joseph, Jaewoo Park, Arezoo Emadi

**Affiliations:** Department of Electrical and Computer Engineering, University of Windsor, Windsor, ON N9B 3P4, Canada; nazemih@uwindsor.ca (H.N.); josep11t@uwindsor.ca (A.J.); jpark@uwindsor.ca (J.P.)

**Keywords:** acoustic gas sensors, carbon nano-tube (CNT) Sensors, electrochemical gas sensors, fiber-optic gas sensors, metal oxide semiconductor (MOS) sensors, organic-based chemiresistive gas sensors, piezoelectric gas sensors, photonic crystal gas sensors, volatile organic compound (VOC), micro-electro mechanical systems (MEMS)

## Abstract

Micro- and nano-sensors lie at the heart of critical innovation in fields ranging from medical to environmental sciences. In recent years, there has been a significant improvement in sensor design along with the advances in micro- and nano-fabrication technology and the use of newly designed materials, leading to the development of high-performance gas sensors. Advanced micro- and nano-fabrication technology enables miniaturization of these sensors into micro-sized gas sensor arrays while maintaining the sensing performance. These capabilities facilitate the development of miniaturized integrated gas sensor arrays that enhance both sensor sensitivity and selectivity towards various analytes. In the past, several micro- and nano-gas sensors have been proposed and investigated where each type of sensor exhibits various advantages and limitations in sensing resolution, operating power, response, and recovery time. This paper presents an overview of the recent progress made in a wide range of gas-sensing technology. The sensing functionalizing materials, the advanced micro-machining fabrication methods, as well as their constraints on the sensor design, are discussed. The sensors’ working mechanisms and their structures and configurations are reviewed. Finally, the future development outlook and the potential applications made feasible by each category of the sensors are discussed.

## 1. Introduction

Gas sensors are critical components of intelligent detection systems, gaining attention due to their broad range of applications such as in the medical field, indoor and outdoor air quality monitoring systems, environmental science, automotive industry, and military [1,2,3]. Gas sensors are commonly categorized under chemical detector branch. They comprise a transducer and an active layer, which converts a desired chemical reaction into a measurable electronic signal such as change in the resistance, frequency, current, or voltage. The performance of such a sensor is evaluated by various parameters including device sensitivity, selectivity, precision, limit of detection (LOD), resolution, accuracy, reversibility, recovery time, and response time. In addition, the device level of miniaturization and power consumption, cost-efficiency, long lifetime, and potential integration with wireless network are considered key factors in the sensor’s implementation in various applications. In recent decades, there have been various studies on gas-sensing technology that established several branches of detection methods with some receiving more attention due to their unique principle of operation, fabrication methods, and prospective applications. This paper provides a review of the recent advancements made in electrochemical sensors, including metal oxide semiconductor and organic-based chemiresistive gas sensors, carbon nano-tube sensors; acoustic gas sensors including quartz crystal micro-balance, surface acoustic wave, and capacitive micro-machined gas sensors; and optical sensors including fiber-optic sensors and photonic crystal gas sensors. The structure and working mechanism of these devices along with their fabrication methods, as well as the recent advances made in the sensors’ design are reviewed and discussed. Furthermore, an outlook of the sensors’ future development and potential applications in various fields are described.

## 2. Electrochemical Sensors

### 2.1. MOS Gas Sensors

Metal oxide semiconductor (MOS) sensor was first introduced decades ago where the resistance of Cu${}_{2}$O was found to be affected by the adsorption of water vapor [1]. However, rapid development of semiconductor sensors started from the result of studies on ZnO and SnO${}_{2}$ thin films, which led to the first commercialized SnO${}_{2}$-based sensors for inflammable gas detection and brought the semiconductor sensors into practical use. Presently, metal oxides play a significant role in many areas of chemistry, physics, and materials science due to their unique structure and advantages such as low cost, short response time, and long lifetime.

#### 2.1.1. Structure and Mechanism

The MOS gas-sensing mechanism is based on the change in the conductivity of the device-sensing component in the presence of reducing or oxidizing gases. In these sensors, the sensing layer is in direct exposure to the target analytes. The interaction between the sensing layer and analytes results in changes in the physical and chemical properties of the sensing material at elevated temperatures [4], leading to a device conductivity change. This conductivity change is due to the variations of the depletion layer at the grain boundaries, which are present at the interconnected metal oxide grains in the active sensing layer of the device. The interaction between active sensing layer and target analytes at an elevated temperature lead to modulations in the energy barriers for free charge carriers, thus leading to a change in the conductivity of the sensing materials.

**Figure 1:**
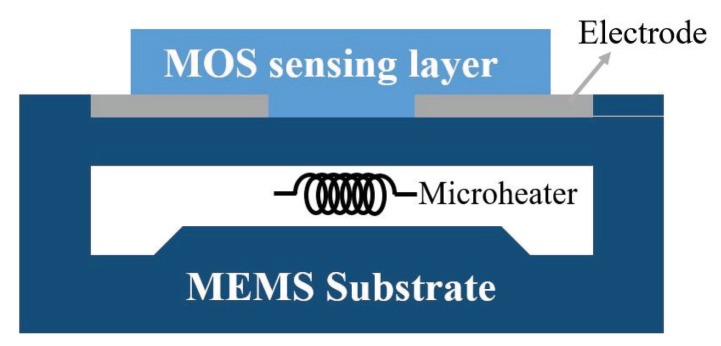
Cross sectional view of a MOS sensor comprising of a set of electrodes, micro-heater, and sensing layer fabricated on a thin suspended membrane using MEMS fabrication technology. The change in the sensing material conductance due to the interaction with analytes is proportional to the concentration of the analytes in the sensor environment.

MOS gas sensors employ specific sensing materials deposited on a set of electrodes along with the required micro-heater that is electrically separated from the sensing element by an insulating layer. Figure 1 illustrates the schematic view of a MOS sensor. Advanced micro-electro mechanical system (MEMS) fabrication technology can be employed to develop MOS sensors on temperature efficient platforms, where the device-sensing area is built on a thin suspended membrane to achieve an optimal thermally isolated structure.

The sensor working mechanism is based on the correlation between the resistance and energy barrier which changes by charge carrier density at the grain boundaries. At high temperatures, oxygen is adsorbed on the surface of MOS. The adsorbed oxygen captures electrons from conduction band, which leads to a change in the charge carrier concentration that affects the resistance of MOS sensing layer. The change in the sensor resistance is directly proportional to the concentration of the exposed analytes [5].

The device sensitivity and properties highly depend on the interaction between the analytes and the sensing layer, therefore, the type of MOS sensor. In general, two kinds of MOS are used: n-type MOS such as TiO${}_{2}$, ZnO, SnO${}_{2}$ and WO${}_{3}$-based sensors and p-type MOS such as NiO, Mn${}_{3}$O${}_{4}$ and Cr${}_{2}$O${}_{3}$-based sensors [6,7,8] with the sensing materials’ thicknesses of ranging from several nano-meters to several tens of nano-meters [5]. The MOS sensor’s sensitivity relies on the thickness of receptor layer, the catalytic metal particles placed in it and the temperature of the receptor layer. The resultant changes in the resistance are different depending on the types of MOS used. The majority charge carriers are electrons in n-type MOS. When it interacts with a reducing gas, an increase in conductivity occurs and if it is an oxidizing gas then depletion of charge carriers takes place, leading to a decrease in conductivity. The majority charge carriers are the positive holes in p-type MOS. The conductivity is increased in the presence of an oxidizing gas as the analytes increases the number of positive charge carriers or holes. On the other hand, if there is a presence of reducing gas, an increase in resistance is observed, where negative charge introduced into the material reduces the positive charge carrier concentration [6].

MOS gas sensors are among the preferred candidates for applications that require detection of low-concentration volatile organic compounds (VOCs) (<ppm levels). In addition, they benefit from measurement simplicity, durability, and ease and low cost of fabrication into small size. MOS sensors with a length of several hundred micro-meters have been fabricated along with the micro-heaters of similar lengths to provide optimum operating temperature [9]. Most of the MOS-based sensors are known to be stable and resistant to poisoning. Consequently, they have become a paramount technology in several domestic, commercial and industrial gas-sensing systems [5]. However, MOS devices have some practical limitations that need to be considered. MOS sensors commonly operate at a high temperature, ranging from 150 ${}^{\circ }$C to 400 ${}^{\circ }$C, which lead to high power consumption. Therefore, this may limit the application of MOS sensors in portable integrated electronics. To lower the operating temperature and obtain a good long-term stability, new porous structures are explored as the next trend of sensing materials development and to develop new solutions to effectively activate metal oxides at low temperatures [10].

#### 2.1.2. Fabrication

Silicon micro-machining technology is the commonly used technique to manufacture MOS sensors. The general fabrication steps include silicon substrate preparation with an insulation layer, micro-heater and sensing layer deposition along with the electrodes [9].

**Figure 2:**
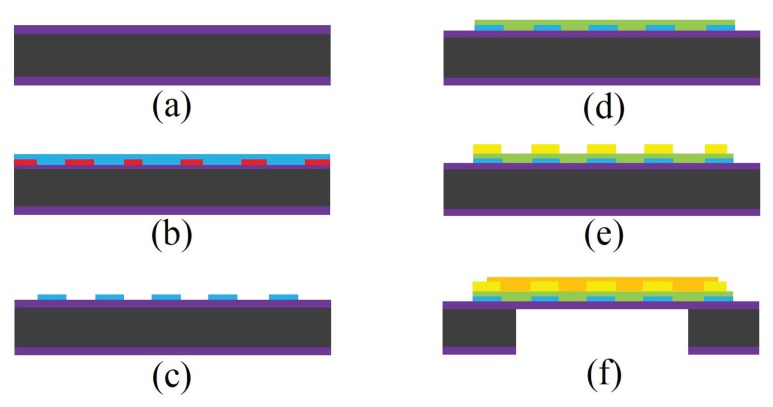
Schematic view of a MOS gas sensor fabrication process (**a**) thermal oxidation of silicon wafer, (**b**) photolithography patterning followed by micro-heater deposition, (**c**) lift-off, (**d**) deposition of a thin SiO${}_{2}$ layer, (**e**) photolithography and pattern transfer of micro-electrodes followed by lift-off process, (**f**) backside etching and deposition of sensing material on the electrodes.

Figure 2 presents a schematic view of MOS fabrication process steps. SiO${}_{2}$ is the commonly used insulating layer that is grown using thermal oxidation to a thickness of couple of micro-meters. The substrate is then etched and thinned by chemical etching or plasma etching. MOS sensors require the use of micro-heaters to initiate the reactions in the sensing layer, which take place at high temperatures. For this reason, a micro-heater is fabricated at the bottom of the device to elevate the sensing area’s temperature. The micro-heater can be fabricated by DC magnetron sputtering of a 10/60 nm thick Ti/Pt metal layer over a micro-heater patterned mask in combination with a lift-off process. To avoid electrical connection between the micro-heater and micro-electrodes/metal oxide sensing layer, the micro-heater is coated with a 300 nm SiO${}_{2}$ insulating layer using e-beam evaporation technique. MOS electrode deposition is done using the same method as of micro-heater and by using DC magnetron sputtering and lift-off process. Electrodes are commonly made of a 10/60 nm thick Cr/Au layer. MOS sensing layer is deposited over the fabricated electrodes, the method of which can vary depending on the employed sensing material. The common SnO${}_{2}$-based MOS sensors use DC magnetron sputtering to deposit 50 nm thick sensing material. In the final step, the backside of the substrate and silicon dioxide is etched by reactive ion etching (RIE) to provide a better temperature isolation and reduce the power consumption [11].

#### 2.1.3. Applications

- MOS sensors for detection of VOCs: A MOS gas sensor in temperature cycled operation can be used to detect formaldehyde, benzene and naphthalene in ppb and sub-ppb concentrations with a varying background of ethanol with concentrations of up to 2 ppm [12]. It has been shown that selective detection of VOCs in the ppb range is possible even with an intensive background of various VOCs. However, sensitivity is reduced compared to the ideal laboratory case and in the absence of background VOCs. For ppb-level VOC detection, MOS gas sensors are designed with temperature cycled operation, which can achieve sufficient sensitivity and selectivity in combination with signal analysis based on pattern recognition. In the temperature cycled operation mode, the heater unit of the sensor is periodically set to different temperature steps and therefore, the MOS sensing layer goes through various states resulting in different level of interaction characteristics between the sensing layer and the target analyte at those specific temperature range [12,13]. Employing the temperature cycled MOS sensor in combination with the pattern recognition techniques can correlate the sensor response to the present analyte in a complex environment by providing an additional identifying parameter. Temperature cycled MOS sensors with detection capability of 100 ppb of formaldehyde and 20 ppb of naphthalene have been reported [12].
- MOS-based sensors with porous sensing layer: To enhance the interaction with the analytes and increase the reaction surface area, the porous structural MOS can be employed that offer high porosity, highly interconnected pore channels, and high surface area and active sites. These porous MOS sensors can be chemically synthesized via soft-templating method and nano-casting strategy [14], which enhances the device performance through facilitating the gas diffusion and improves the sensor’s sensitivity, response and recovery time, and selectivity. The tunability of this synthesis approach provides a potential to develop porous MOS sensors with various compositions, where pore size, film thickness, temperature, and humidity are the factors that affect sensing performance [15].

### 2.2. Organic-Based Chemiresistive Gas Sensors

An emerging class of electrochemical sensors is represented by chemiresistors sensors. They operate based on the change in the electrical resistance of the materials due to their chemical interaction with the analytes. These devices can use various sensing materials such as conductive polymers, organic semiconductors, and carbon-based materials.

#### 2.2.1. Structure and Mechanism

Compared to MOS sensors, organic-based chemiresistor gas sensors benefit from a relatively simple configuration where the need for a micro-heater may be avoided. The geometry of these sensors consists of a sensing material that bridges the gap between two electrodes, deposited on a thin insulation layer such as SiO${}_{2}$ on a silicon substrate.

**Figure 3:**
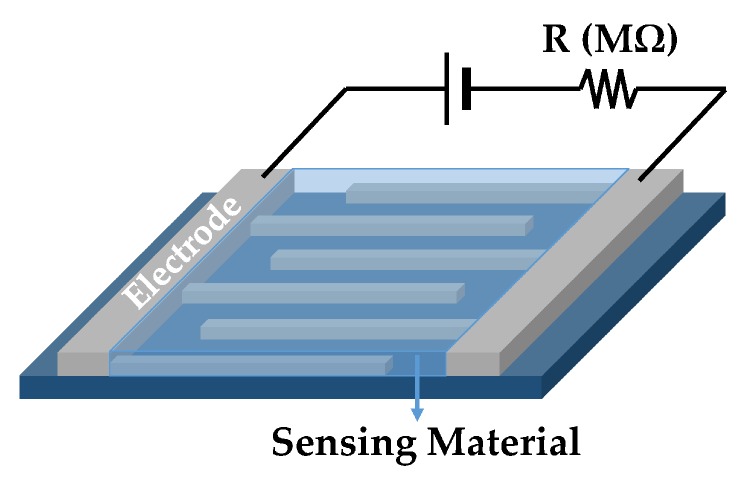
Schematic view of a chemiresistor sensor that measures the sensing material resistance changes between the two interdigited electrodes when exposed to the desired analytes.

Organic-based chemiresistive gas sensors measure changes in the electrical resistance of the sensing material in response to the changes in the sensor environment. When the sensor is exposed to certain analytes, a direct interaction occurs between the sensing material and the analytes that results in a change in the sensing material properties, such as an increase in the material volume or a reduction in material conductivity [16]. The amount of sensor resistance change can be correlated with the analyte properties and concentration. The mechanism of interaction between the analytes and the sensing material defines the level of material properties changes. This can vary depending on the employed materials, e.g., highly sensitive nano-structured dye-doped polypyrrole (PPy) electrosynthesis has been fabricated on gold interdigitated electrodes (IDE) with a reported detection limit and a dynamic range of 0.2 ppb and 9.7–827 ppb, respectively for 2,4,6-Trinitrotoluene (TNT) detection [17]. The electrodes are commonly designed as an interdigitated structure as shown in Figure 3. IDE configuration maximizes the contact area between the electrodes, the sensing materials, and the analytes. The two electrodes are typically connected to an external data processing unit to analyze the physical and chemical change in sensing film [18].

Conducting polymers or insulating polymers that are made conductive are commonly used in chemiresistive gas sensors, e.g., polyaniline (PANI), poly (3,4-ethylene-dioxythiophene) (PEDOT), polypyrrole (PPy), polythiophenes (PTs) [18,19]; inorganic metal oxides materials that provide similar working mechanism as MOS sensors as discussed in the previous section separately for its popularity; and metal nano-particles such as gold nano-particle (AuNP) where the sensor consists of an interdigitated micro-electrode with deposited gold nano-particles.

Polymer-based sensors are the most commonly used type of chemiresistor sensors. They can be divided into five main categories based on the sensors output: conductometric devices where electrical conductivity changes are measured; potentiometric sensors where changes in the chemical potential without current flow are detected; amperometric sensors where measurement of the current generated by the redox reaction of VOCs at the sensing electrode is detected; colorimetric detectors where changes in optical absorption are measured; and gravimetric sensors where change in the polymer weight as a result of VOC–polymer interaction is measured [20].

Compared with MOS sensors, polymer-based sensors offer several advantages such as a high sensitivity and short response time. Moreover, array of sensors can be fabricated using different conducting polymers to improve the system selectivity. Unlike MOS sensors, a promising characteristic of chemiresistor polymer-based sensors is their key ability to operate at the room temperature condition. These polymer-based sensors can still detect many VOCs such as toluene and benzene that are not chemically reacting with the sensing material at the room temperature through measuring the amount of polymer swelling when exposed to such environment [21]. In addition, they benefit from low-cost MEMS fabrication technique and simple and portable structures as well as low energy consumption [22]. However, polymer-based sensors are considered to be temperature dependent [23] with short lifetime. Moreover, depending on the selected organic sensing material, the sensitivity of these sensors can be influenced by the saturating effect of some VOCs and humidity [16]. In addition, the sensor output often exhibits baseline drift and longer response time in comparison with other chemical sensors. To eliminate the environmental temperature fluctuation dependency, these sensors can be fabricated on temperature insensitive membrane that incorporates micro-heaters using MEMS fabrication technology [24].

#### 2.2.2. Fabrication

Chemiresistive gas sensors structure consists of a sensitive element and a pair of electrodes fabricated on a silicon wafer with an insulating layer such as SiO${}_{2}$. An SiO${}_{2}$ insulating layer can be grown by thermal oxidation or deposited using e-beam evaporation process to prevent the electrical connection between the substrate and the sensing material. The electrodes are then deposited and patterned followed by the sensing layer deposition to prevent any damage to the sensing layer during the deposition process. Depending on the chosen sensing layer, it can be deposited using spin coating, spray coating, dip coating, or drop casting techniques [17].

#### 2.2.3. Applications

Various polymer-based sensors have been fabricated for different applications such as healthcare monitoring and environmental pollutants measurement [25].

- Capped nano-particle sensors for VOCs detection: Molecularly capped nano-particles have been intensively investigated as the chemiresistor sensing materials to detect various VOCs. Chemiresistor arrays have been fabricated with cross-linked Au nano-particle thin films with subtle structural differences. These chemiresistive sensors detect VOCs and breath biomarker under ambient conditions. The nano-particle composition and size are the key parameters in designing the sensor array with desired sensitivity, selectivity, and stability. This sensor configuration has been used for breath recognition of lung cancer patients. Nano-structured sensor arrays have shown high sensitivity and selectivity in detecting mixtures of VOCs with a LOD as low as 20 ppb [26].
- Polypyrrole sensors for detection of explosive gases: Chemiresistor gas sensor based on sulfonated dye-doped modified conducting polypyrrole (PPy) film has been designed and fabricated for highly sensitive detection of 2,4,6-trinitrotoluene that is one of the most commonly used explosives. The sensor uses electro synthesis of PPy on Au-IDEs in the presence of the sulfonated dyes and exhibits high sensitivity and good selectivity to TNT with no response to other related gases and with a LOD and linear dynamic range of about 0.2 ppb and 10–800 ppb, respectively [17].

## 3. CNT Gas Sensors

Advanced micro- and nano-fabrication technology have led to the introduction of carbon nano-tubes (CNTs) sensors [27]. A unique characteristic of these sensors is the use of CNT as the sensing material where their properties depend on their shapes. CNTs are categorized in single-walled carbon nano-tubes (SWCNT) and multi-walled carbon nano-tubes (MWCNT). SWCNTs consists of one layer of CNTs while MWCNTs have different layers of CNTs inside each other. The unique properties of CNTs come from the high aspect ratio with very strong intermolecular bonds and low density, resulting in an enhanced sensitivity, low detection limit, and fast response time [28].

### 3.1. Structure and Mechanism

Due to the unique properties of CNT sensors, they have been employed as a popular candidate in gas-sensing applications. Based on their principle of operation, these sensors can be divided into categories of gas sorption, gas ionization, capacitive and resonant frequency gas sensors:

- Chemiresistive gas sorption CNT sensors: In these sensors exposing CNTs to the target gas results in charge transfer between the CNT and the gas. This phenomenon results in a change in conductivity of the CNT sensing material. The change in the device conductivity is correlated with the gas properties and concentration. In these sensors, recovery time can be improved by heating the sensing film [27]. The sensing property of sorption-based CNT gas sensors can be modified by using different chemical functional groups such as oxygen on the surface of CNTs where they can lead to selective interaction to desired analytes such as hydrogen-containing molecules. However, this can decrease the accessibility of the analytes to the CNT surface, hence reducing the sensitivity [29]. Common disadvantages of these sensors are long recovery time, irreversibility of CNT conductivity and decreased sensitivity for low gas energy levels [27].
- Gas ionization CNT sensors: High aspect ratio of CNTs provides an ideal geometry for creation of an electrical field by applying voltage. In ionization CNT gas sensors, CNTs are used for both anode and cathode electrodes to create electric field [30]. Analytes is ionized to be in plasma state by the accelerated electrons from the electrode. The ionization energy and the current though the plasma can be measured for identification of gas properties and concentration. This mechanism is useful for detection of gases with low sorption energy. Common gas ionization sensors are bulky with high energy consumption level; however, the use of CNTs can reduce the size and ionization energy of gas significantly due to the easier ionization enabled by CNT’s sharp tip structure and low work function [30,31].
- Capacitive-based and resonant frequency CNT sensors: CNTs can be used as the sensing element of capacitive-based gas sensors. In this structure, one plate of the capacitor is silicon and the other plate is made of is CNT coated silicon. By applying a voltage across the capacitor, CNT creates a high electric field which results in polarization of the gas molecules, and therefore, a change in the capacitance. The shift in the sensor’s capacitance is due to the dielectric constant change of CNT which is correlated with the target VOC concentration. This dielectric change of CNT sensor can also be used in a resonance frequency sensor configuration which measures the frequency shift associated with the gas properties and concentrations [32].

### 3.2. Fabrication

Advanced micro- and nano-fabrication technology are used to fabricate CNTs with tube diameter of nano- to micro-meter range and a length of tens of micro-meter. CNTs were traditionally fabricated using pulsed laser ablation (PLA) or arc discharge. However, more advanced CNTs are developed by employing chemical vapor deposition (CVD) technique where the fabrication temperature remains below 800 ${}^{\circ }$C [33,34].

**Figure 4:**
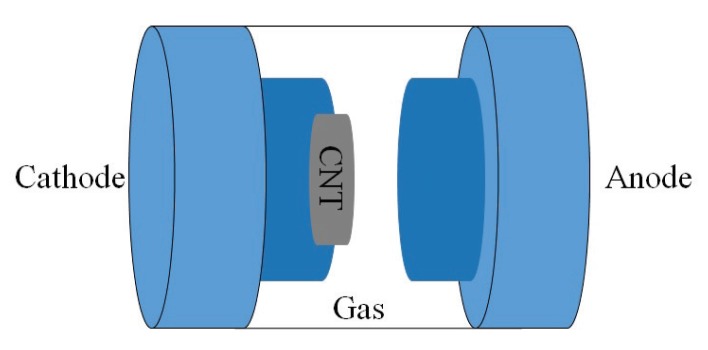
Fabricated CNT using an arc discharge method. The chamber with graphite electrodes is filled with helium, hydrogen, or methane. The high temperature causes the graphite to sublimate and move towards the cathode and create a CNT layer on it.

**Figure 5:**
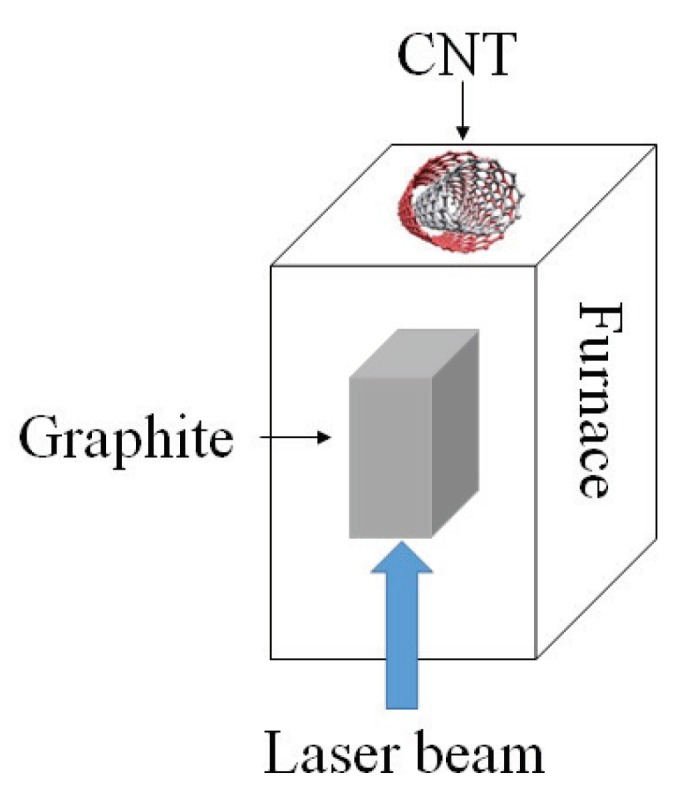
Schematic diagram presenting the pulsed laser ablation method. A graphite target in the reactor evaporates using a laser beam and the CNT layer forms on the surface at the end of the chamber.

**Figure 6:**
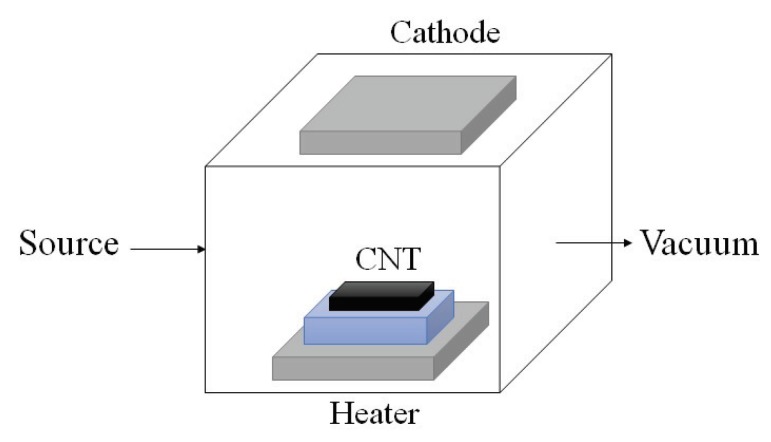
Schematic diagram of a PECVD technique where CNT layer is being created on the substrate in a vacuum chamber while a strong electric field generates plasma. The created field causes the nano-tube layers to grow along the electric field and perpendicular to the substrate.

- Arc discharge technique: Since the synthesis temperature is above 1700 ${}^{\circ }$C, arc discharge technique has been reported to create less defects in the CNTs. In this method, fabrication is processed in helium, hydrogen, or methane-filled chamber containing graphite electrodes as shown in Figure 4. By applying the voltage, the electrode evaporates in gas and form CNTs on the other electrode [33,34].
- Pulsed laser ablation: PLA method has been used to produce SWCNTs of high quality and purity. The procedure is very similar to the arc discharge technique except that the energy is provided by a laser source. The laser is introduced onto the graphite layer which contains cobalt or other catalysts. A schematic diagram of PLA method is shown in Figure 5.
- Chemical vapor deposition: CVD has been proposed as an alternative to conventional CNT fabrication techniques due to its controllable process as well as high purity of its product.
  There are different CVD methods that have been used for CNT synthesis fabrication, such as catalyst CVD (CCVD), plasma enhanced CVD (PECVD) shown in Figure 6, radiofrequency CVD (RF-CVD), micro-wave plasma CVD (MPECVD) and water and oxygen assisted CVD. These days, CCVD and PECVD techniques are standard methods to synthesize CNTs, since they provide purer CNTs while using low temperature processes.

### 3.3. Application

- Gas sorption CNT sensors for NH${}_{3}$, NO${}_{2}$ and organic compounds detection: Monolayer CNTs have been used for detection of NH${}_{3}$ and NO${}_{2}$ in mass adsorption principle. Limit of 44 ppb and 262 ppb have been reported for detection of NO${}_{2}$ and nitrotoluene, respectively [27]. Long recovery time up to 10 h is reported due to the very strong molecular bonds between some analytes and carbon, which is improved to 10 min by applying ultra-violet (UV) radiation to break bonds [27]. In addition, CNTs have been used in sensors employing field effect transistors (FET) to improve selectivity between NO${}_{2}$, CO${}_{2}$, CO, O${}_{2}$ and H${}_{2}$ [35,36].
- Gas sorption CNT sensors for C${}_{2}$H${}_{2}$ and H${}_{2}$O detection: CNT sensors have difficulty in detecting gases such as CO, water vapor and bimolecular gas since they are limited to detect molecules with high bonding energy and ability to transfer charges to the nano-tubes. To overcome this disadvantage, various doping of CNTs have been proposed. In mass adsorption gas sensors, CNTs doped with nitrogen and boron are reported to detect C${}_{2}$H${}_{2}$, H${}_{2}$O and NO in a low concentration at room temperature. Boron doped CNTs have good sensitivity for ethylene (C${}_{2}$H${}_{2}$), while nitrogen doped CNTs are sensitive to NO${}_{2}$ and CO [37].

## 4. Acoustic Gas Sensors

Acoustic sensors respond to the absorption of analytes in the sensing film and sense gas molecules that can be immobilized on the surface of the device. In these sensors, the analytes which provide better chemical interactions can be detectable in lower concentration levels. Acoustic sensors with ppm to ppb minimum detection level have been reported [38]. However, a main reported problem associated with the acoustic sensors is their limited selectivity that is associated with the common employed sensing materials. Several methods have been reported to increase these sensors’ selectivity such as using an array of sensors with different chemical interfaces and employing pattern recognition algorithm to identify analytes, as well as adding chromatographic column to the sensor to separate different absorbed analytes. Several acoustic gas sensors have been proposed and investigated such as quartz crystal micro-balance (QCM), surface acoustic waves (SAW), flexural plate wave (FPW) and thin rod sensors. QCM and SAW sensors are actively being researched and are introduced in this section. Capacitive micro-machined ultrasonic transducer (CMUT) gas sensor has also been introduced, which uses mass changes of thin sensing film.

### 4.1. QCM Gas Sensors

When Quartz or a similar piezoelectric crystal are subjected to the electrical or mechanical stress, a voltage proportional to the amount of the stress is produced in the piezoelectric crystal. The oscillation frequency of the quartz crystal is dependent on the change of mass in the surface of the crystal, which is used as the principle of operation of QCM [39].

#### 4.1.1. Structure and Mechanism

**Figure 7:**
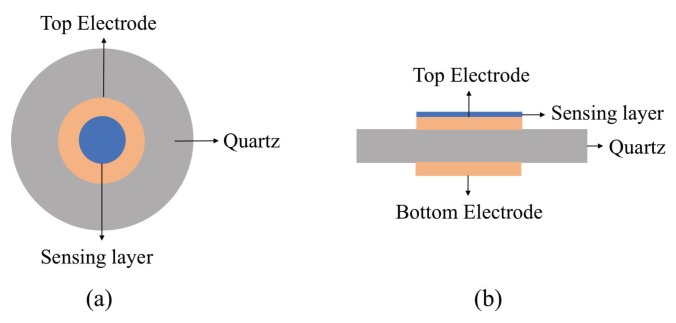
Schematic view of a typical QCM sensor (**a**) top view and (**b**) side view. The quartz crystal resonator is sandwiched between two gold electrodes (yellow). The thin film sensing layer on the top of QCM structure (blue) attracts analytes, changing the mass, measuring through sensor resonance frequency shift.

QCM sensors uses a piezoelectric quartz crystal that is sandwiched between two electrodes and coated with the sensing material as presented in Figure 7. The sensing electrode placed on the top side of the quartz and a reference electrode is deposited on the opposite side. The sensing material is coated at the center of top sensing electrode which is exposed to the analytes. QCM sensors detects the mass change of the sensing layer by measuring resonance frequency shift of the quartz. When external electric field is applied to the quartz via the two electrodes, an asymmetry in dipole moments of the crystal structure is created, resulting in quartz deformation. Therefore, this property can be used to create standing wave between the two electrodes by applying alternating current at the device resonant frequency. When the mass on the surface of the crystal changes due to the analyte adsorption, the crystal resonant frequency changes, which can be correlated with the gas properties and concentrations as shown by Equation (1). This resonance frequency change caused by surface mass changes are shown by [39]:

(1) Δm=−C·Δf

(2) $$\Delta f=\frac{-2f_{0}\cdot 2\rho_{s}}{\sqrt{\mu_{q}\cdot \rho_{q}}}$$

where $f_{0}$, $\rho_{s}$, $\mu_{q}$, and $\rho_{q}$ are the reference resonant frequency, the surface mass density, shear stiffness, and density of quartz, respectively. This only applies to the elastic subjects such as metallic coating, metal oxides, and thin adsorbed layers that do not dissipate energy during the oscillation. Inelastic subjects such as cells, polymers, and bio-molecular systems may exhibit energy loss due to the viscous damping during the resonant frequency oscillation of the crystal [40]. Due to the significant deformation of the crystal, the choice of sensing material is important. Polymers are commonly good candidates for sensing material that can undergo such deformation across the film, despite energy dissipation.

The mass sensitivity of the QCM is dependent on the thickness of the crystal. The shear stiffness of the crystal and the resonant frequency are inversely proportional to each other based on Equation (2). Therefore, to achieve high resonance frequency in QCM, the thinner quartz resonator is required to lower $\mu_{q}$, the shear stiffness, and enhance sensitivity. However, the use of thin film can be limited due to the difficulty in fabrication process.

#### 4.1.2. Fabrication

**Figure 8:**
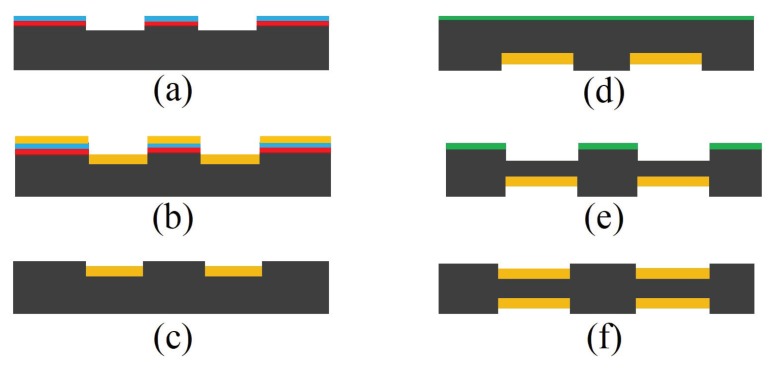
Schematic view of a QCM sensor fabrication steps (**a**) AT-cut quartz crystal of thickness of approximately 168–330 μm is polished, followed by deposition of chromium layer, photolithography and wet etched steps, (**b**) Au layer of thickness of approximately 100–200 nm is deposited by electron beam evaporation, (**c**) gold electrodes are formed by lift-off process, (**d**) nickel layer is deposited using sputtering technique, (**e**) nickel layer is patterned and DRIE is carried out to transfer the pattern to the quartz, (**f**) electrodes are deposited using electron beam evaporation method followed by the lift-off process.

QCM sensor is a thickness-shear mode device, where crystallographic orientation can affect the performance of the sensor. Therefore, the resonant frequency of the crystal depends on the angle which the quartz wafer was cut from the crystal. Figure 8 illustrates fabrication steps of QCM sensors. Depending on the target frequency, QCM can commonly be fabricated on a quartz with a thickness ranging from 168 μm to 330 μm and a diameter of about 5 mm to 25 mm. Multichannel QCM sensors have been fabricated with diameters ranging from 0.05 to 1.0 mm on a quartz plate with dimension of 10 mm × 12 mm and distance between the resonators of about 1 mm [41]. Chemically stable metals such as gold and platinum are commonly used as the QCM electrodes. Anisotropic inductively coupled plasma reactive ion etching (ICP RIE) method can be used to etch quartz. A thin Cr film can be used as the mask and deposited using sputtering technique at room temperature followed by photolithography and wet etching of the quartz plate in HF solution of about 16% Pt/Ti or Au layers are then deposited using electron beam evaporation. Au electrodes can be formed using lift-off process. Micro-machining techniques have also been used to fabricate arrays of bulk wave resonator sensors. To have a hybrid sensor array, they can also be combined with oscillator circuitry fabricated on a silicon [42]. High frequency bulk wave resonator has also been formed by using piezoelectric films and bulk Si micro-machining techniques. While typical resonator sensors provide frequencies of 5–10 MHz, the resonant frequency of approximately 1 GHz have also been developed, resulting in high mass sensitivity [43].

#### 4.1.3. Applications

- Calixarene coated QCM for VOCs detection: QCM sensors with calixarene coating or calixarene derivatives have been employed for detection of analytes such as alcohols, halocarbons, esters, ethers, chemical warfare agents, and toxic gases. Calixarene modified QCM sensors exhibit strong sensing ability to methylene chloride emissions with a LOD of about 54 ppm. The cyclic structures, hydrogen-bonding capabilities, and highly organized properties of the calixarene derivatives play a key role in VOC detection. However, QCM sensing properties are affected due to the random arrangement of calixarene derivatives’ molecules on the surface of the crystal [44].
- PANI-ES coated QCM for vapor detection: QCM can be fabricated based on dip coated polyaniline emeraldine (PANI-ES) salt thin films. In these devices, three different acids thin films such as hydrochloric acid (HCl), dodecylbenzene sulfonic acid (DBSA) and 1,5-naphtalene disulfonic acids (1,5-NDSA) are doped on the AT-cut 10 MHz QCM electrode. These sensors exhibit a frequency shift linear to both the vapor concentrations in part per million (ppm) and the sensing film thickness in nano-meter (nm). The frequency changes in these sensors are mainly due to the electrostatic interactions between the dopant agents within PANI-ES films and the vapor molecules. PANI-DBSA films show a highly sensitive of ~7 Hz/ppm and selectivity to para-xylene over toluene and benzene with a LOD of 3 ppm. They exhibit a relatively short recovery time of less than 3 min and an acceptable sensitivity in the presence of humidity interference [44].
- Ultrasensitive PPy-BPB and -4BP-based QCM for gas detection: Polypyrrole-bromophenol blue (PPy-BPB) nano-structure-based QCM has been developed for the detection of very small trace amounts of nitro-explosive vapors. This ultrasensitive and selective QCM sensor uses PPy-BPB compound in nano-sphere and nano-rod forms on the gold electrode. At room temperature the sensors are stable, reversible, and exhibit fast response time. PPy modified QCM sensors can be doped with various bromine containing anion dopants to improve the sensor performance. The enhanced sensitivity of this sensor towards nitro-explosives is related to the non-covalent interaction of halogen-nitro synthons between the bromine atoms and nitro-explosive groups as electron deficient acceptors as well as the partial charge transfer interaction between the nitro-explosive groups and electron rich polymer film [45].

### 4.2. SAW Device

The first generation of SAW gas sensors were introduced decades ago with an ultra-high resonant frequency of 400 MHz [46]. These sensors detect environmental changes based on the change in the physical properties of the surface waves and amplitude. The measured concentration of detected analytes has been reported to be in picogram scale. In general, high sensitivity, short response time and reversibility are reported as advantages of SAW gas sensors beside being applicable in wireless technologies.

#### 4.2.1. Structure and Mechanism

**Figure 9:**
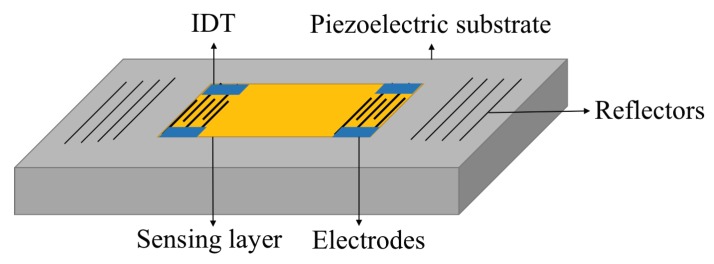
Schematic view of a SAW gas sensor consists of two arrays of reflectors.

SAW sensors include two SAW reflector arrays on the crystal substrate separated by a cavity where a pair of electrodes is located. The sensor length can be up to several millimeters. A schematic diagram of a SAW gas sensor is illustrated in Figure 9.

The reflector arrays consist of metal strips with a half-wavelength width. A part of the wave’s energy reflects on the spacing between the strips where it gives an almost a full reflection. The reported operating frequencies for the sensors with GaAs substrate are as high as 100–500 MHz, which results in complexities in the sensor design and fabrication [47]. SAW sensors can be electrically excited and detected in a piezoelectric substrate using a transducer. In this configuration, and unlike bulk quartz resonators, the frequency of SAW sensors does not depend on the wafer thickness. The operating frequency in such resonators is calculated based on the transducer periodicity, f=ν/λ, where ν and λ are the propagation velocity and acoustic wavelength, respectively at the transducer center frequency. By changing the surface mass when exposed to the analytes, the propagation speed changes which causes a frequency shift from the operating frequency. The frequency is closely related to the sensor response which can be affected by other factors such as change in viscoelasticity and electrical conductivity due to the absorbed analytes [47].

#### 4.2.2. Fabrication

The choice of SAW sensing materials, electrodes, and substrates depend on the target wave propagation properties. The most common materials used as substrate in SAW sensors are LiNbO${}_{3}$, GaPO${}_{4}$, LiTaO${}_{3}$, silicon and quartz which are functionalized by a sensing material such as piezoelectric zinc oxide (ZnO) and aluminum nitride (AlN) [46,47]. GaAs substrates have also been used without the need for a piezoelectric film. Aluminum electrodes and reflectors have been widely used due to its acoustic impedance similarity to the common SAW substrates. E-beam lithography and lift-off processes are commonly used to fabricate SAW gas sensor on gallium orthophosphate (GaPO${}_{4}$) substrate. GaPO${}_{4}$ is one of the preferred piezoelectric materials for substrates due to its high thermal stability and since the device operates at temperatures up to 930 ${}^{\circ }$C. In this high temperature sensor, platinum has been used as electrodes because of the high melting temperature. To have a good adhesion, a zirconium or titanium layer has been used between the substrate and electrodes [48].

#### 4.2.3. Applications

- Polymer-based SAW for biomarker and VOCs detection: SAW gas sensors can employ various polymers as their sensing element that can react to different analytes such as biomarkers associated with lung cancer. However, many of these polymers respond to the presence of more than one analyte. The number of chosen sensing materials and their properties are designed according to the type of biomarkers that need to be identified. Pattern recognition and neural network techniques are employed to discriminate various chemical analytes by analyzing signal obtained from these sensors with different sensing material [46].
- Palladium- and CuPc-based SAW for hydrogen detection: Palladium has been used as the sensing material on a SAW sensor to detect hydrogen. Absorbing and desorbing hydrogen molecules result in a change in density and electrical conductivity of the sensing material. Copper phthalocyanine (CuPc) has also been used in SAW system for hydrogen detection. It has been shown that CuPc layer alone is not sensitive enough to hydrogen, which required high operation temperature of more than 70 ${}^{\circ }$C. This operating temperature can be lowered by using CuPc or Pd thin film as sensing layer down to room temperature. In this design, the change in the sensor’s output is mainly due to the change in the electrical conductivity rather than the mass change of the sensing layer [49].

### 4.3. CMUTs

CMUTs have been introduced as an alternative to the conventional piezoelectric transducers with improved properties such as higher bandwidth and better acoustic matching [50]. CMUT configuration can also be employed in the gas sensor field by functionalizing a sensing material to detect VOCs and different gases, which provides a wide range of application. In addition, good sensitivity, low LOD, reversibility, and high-quality factor are reported as the advantages of these sensors [51].

#### 4.3.1. Structure and Mechanism

**Figure 10:**
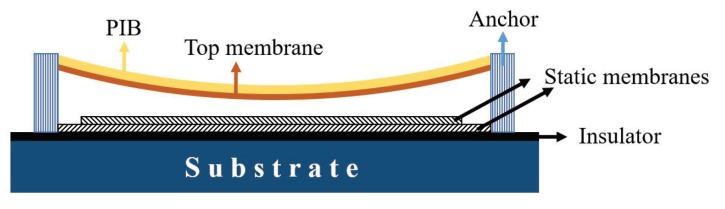
Schematic view of a CMUT sensor where the top flexible membrane is coated with a thin layer of PIB.

CMUT gas sensor consists of a thin flexible membrane coated with a sensing material suspended over a fixed bottom electrode. In this design, the top membrane and the bottom electrode act as a capacitor where changes in the device-sensing material can influence the device capacitance [52]. A schematic view of a coated CMUT sensor with polyisobutylene (PIB) is shown in Figure 10. This device employs stack of polysilicon and gold as the membrane materials and is fabricated on a silicon nitride coated silicon substrate. Polymers are widely used as the CMUT sensing material to absorb the analytes. By exposing the polymer to the gas, analytes are absorbed by the sensing material which results in a change in the mass of the flexible membrane. This mass change results in a center resonant frequency shift, which is correlated with the analyte concentration. The relation between the center resonant frequency, material properties and structural dimensions are determined by Equation (3):

(3) $$f_{0}=0.47\frac{t_{m}}{r_{m}^{2}}\sqrt{\frac{E_{m}}{\rho (1-\nu^{2})}}$$

where $t_{m}$, $r_{m}$, $E_{m}$, ρ and ν are the thickness, radius, Young’s module, density, and Poisson’s ratio of the membrane, respectively [51]. CMUT sensors require a relatively high DC bias voltage known as pull-down voltage to create an electrostatic force across the cavity and bring the top membrane to an optimal point defined by the cavity height and membrane physical properties [50]. In this configuration, several parameters affect the sensitivity of the sensor such as the structural and material properties, radius, membrane thickness, sensing material initial thickness, cavity height, and pull-down voltage. It has been shown that smaller radii and membrane thicknesses provide higher mass sensitivity. CMUT chemical sensor with mass sensitivity of 130 zg/Hz/μm${}^{2}$ have been developed [51]. In addition, since the top membrane of a CMUT sensor is backed with vacuum this structure provides a lower energy consumption and higher quality factor [51]. Compared to conventional capacitive gas sensors such as micro-machined cantilever-based sensors, the analytes cannot reach the cavity underneath the vibrating membrane and therefore the sensor resolution is enhanced [53]. CMUT gas sensors has similar disadvantages as chemiresistive sensors including poor selectivity and baseline drift associated with the common employed sensing materials. These drawbacks, however, can be addressed through fabricating sensor arrays using different sensing materials.

#### 4.3.2. Fabrication

**Figure 11:**
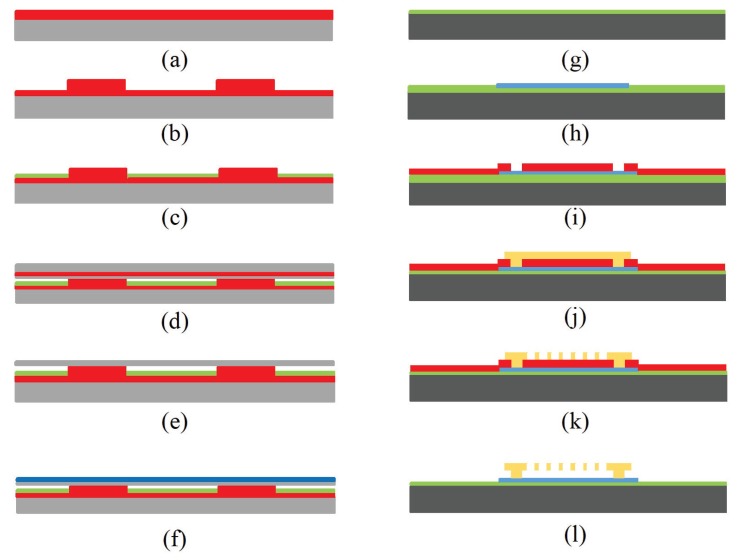
Schematic view of CMUT fabrication process using fusion bonding methods ((**a**) wet oxidation of a highly doped silicon substrate followed by a lithography step, (**b**) growing SiO${}_{2}$ by wet oxidation to further raise the bottom electrode, (**c**) oxide etch followed by the thermal oxidation of a thin SiO${}_{2}$, deposition of Si${}_{3}$N${}_{4}$, patterning and etching, (**d**) local oxidation to create anchors, (**e**) wafer bonding SOI wafer in vacuum followed by annealing, (**f**) removing carrier wafer and buried oxide layer) as well as sacrificial technique ((**g**) deposition of Si${}_{3}$N${}_{4}$ insulation layer, (**h**) deposition of polysilicon bottom electrode, (**i**) deposition of SiO${}_{2}$ sacrificial layer followed by lithography patterning, (**j**) deposition of the top membrane polysilicon layer, (**k**) patterning top membrane to create sacrificial release holes, (**l**) release of the top membrane using wet etching).

Advanced micro-machining techniques can be used to fabricate CMUTs. Individual CMUT cells with radius ranging from 1 μm to several mm and cavity height of tens of nano-meters can be fabricated on the same substrate to form a 1- or 2-D sensor array in millimeter scale [52,53,54]. The two common micro-machining methods are sacrificial and wafer bonding techniques [54,55,56], shown in Figure 11, where the main difference between these two techniques is the method to create the cavity between the two membranes.

In sacrificial method, a sacrificial oxide layer is deposited on the fixed bottom electrode which later forms the cavity followed by the deposition of the top membrane material. The sacrificial layer is then etched using wet etching process and through several release holes on the top membrane or designated channels [57]. A more advanced technique to fabricate a CMUT is a wafer bonding method. In this technique, cavities are patterned and created on a handling wafer that bonds to a second wafer with the deposited top membrane material in vacuum and at high temperatures. Second wafer substrate is then etched or polished to leave a thin membrane suspended over the cavity. SOI wafer can also be used as the second wafer, where the top silicon layer acts as the membrane material and oxide as the etching stop when removing the bulk silicon substrate. Sacrificial method has been widely used for CMUT fabrication due to ease of fabrication and lower cost and complexity; however, wafer bonding method provides several advantages such as elimination of releasing holes that improved device efficiency as well as ability to precisely control and optimize cavity and membrane thicknesses. Moreover, the bonding technique provides capability to fabricate large membranes due to the stress-free processes; however, the quality of bonding is largely affected by the smoothness of the contact surface. The top membrane is coated with the sensing material for CMUT configuration to operate as a sensor. Since the polymers are coated as a sensing layer, they can be deposited on the top membrane by dip coating or spin coating on cleaned CMUT devices [58].

#### 4.3.3. Application

Using various sensing materials make CMUT sensors applicable in different fields such as biomedical and environmental fields.

- CMUT sensors for dimethyl methylphosphonate (DMMP) detection: Employing a very thin layer of DKAP polymer, CMUT sensors have been reported to detect DMMP, a simulant for sarin gas, with a good selectivity and a sensitivity of 48.8 zg/Hz/μm${}^{2}$ [59]. In addition, polyisobutylene (PIB) coated CMUT sensor is also reported to detect DMMP with a sensitivity of 130 zg/Hz/μm${}^{2}$ [51] with a minimum LOD for DMMP of 16.8 pptv [53].
- CMUT sensors for carbon dioxide detection: CMUT sensors employing different materials such as polyimide, amine-bearing functional groups and quinidine can be fabricated as a highly sensitive CO${}_{2}$ detector. A CO${}_{2}$ sensitivity of 1.06 ppm/Hz at 50 MHz and a resolution of 4.9 ppm in the ambient temperature has been reported with consideration of other influencing parameters such cross sensitivity with water vapor, sensor repeatability and regeneration [58].

## 5. Optical Gas Sensors

Optical sensors operate based on control, manipulation, or detection of the propagation of light in an active area where the detection of photons directly results in electronic signals. Two commonly used optical sensors, fiber-optic gas sensors and photonic crystal gas sensors, are discussed.

### 5.1. Fiber-Optic Gas Sensors

Fiber-optic sensors have been proposed as potential candidates for environmental monitoring applications. Fiber-optic sensors can be used in an array where individual sensing device has different selectivity with a pattern recognition system to differentiate various analytes. Fiber-optic sensors may have limitation in miniaturization due to the size of optical fiber itself. However, they are reported to be high in selectivity, sensitivity and stability [60].

#### 5.1.1. Structure and Mechanism

**Figure 12:**
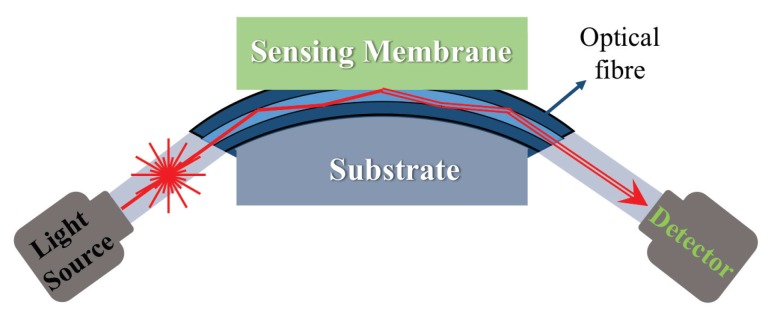
Schematic view of fiber-optic sensors. The probe light enters the optical fiber with initial wavelength $\lambda_{0}$ and is introduced on sensing material. The wavelength of light shifts under the influence of change in optical or optoelectronic properties of sensing film by analytes.

Fiber-optic sensors are composed of sensing layer, optical fiber and the substrate as shown in Figure 12. The polished optical fiber is held on the substrate to partially expose the sensing layer to light. The sensing membrane is placed above the polished fiber where the interaction between the analyte and the sensing layer occurs that produces physical and chemical changes such as in refractive index. Therefore, fiber-optic sensors can detect analytes that create a measurable optical or optoelectrical changes in the sensing layer [61].

The working mechanism of fiber-optic sensors can be explained by pulse width modulation (PWM) as well as analytes-sensing layer interaction. In PWM-based fiber-optic gas sensors the pulse width changes with the change in properties of sensing layer when exposed to desired analytes [61]. This technique enables detection of small changes in light associated with the light pulse amplitude and fall time. The sensing layers for fiber-optic sensors are prepared using dyes such as solvatochromic dyes into polyvinylpyrrolidone (PVP) or polyvinyl chloride (PVC). The refractive index of the sensing layer changes when it is exposed to analytes, due to the charge transfer character of the dye [62].

#### 5.1.2. Fabrication

The fiber-optic sensors are made by attaching an optical fiber onto substrate followed by the deposition of a sensing material. A curved V shaped groove with a desired radius of curvature is created on the quartz substrate to host the optical fiber [63]. The sensing membrane is the deposited on the top of the structure using different methods depending on the employed sensing materials for a specific application. Polymers have been commonly used as the sensing materials in fiber-optic sensors. In case of polymeric sensing layer, the polymer film is dissolved in a solvent and deposited on the optical-fiber-held substrate by spin coating, dip coating, or spray coating [61].

#### 5.1.3. Applications

- Cholesteric liquid crystal film coated fiber-optic sensors for VOCs detection: A cholesteric liquid crystal film (CLCF) coated side polished fiber (SPF) has been used for VOC sensing where an increase in VOC concentration on CLCF results in an increase in the pitch of the resultant light. This creates a blue shift of the resonant dips which can be correlated with the exposed VOCs. The sensitivities of the CLCFC-SPF have been reported to be 7.08 nm.L/mmol, 3.46 nm.L/mmol and 0.52 nm.L/mmol for tetrahydrofuran, acetone, and methanol gas, respectively, where the sensitivity of CLCFC-SPF increases with the molar mass of the VOCs [64].
- ZnO nano-particle-based fiber-optic gas sensor: ZnO nano-particles coated fiber-optic sensors have shown concentration selectivity dependency for acetone, ammonia, and ethanol. The ZnO nano-particles show good sensitivity towards ammonia at low concentrations up to 150 ppm and acetone at high concentrations above 150 ppm due to the enhanced catalytic reactivity of acetone at high concentrations [65].
- ZnO thin film-based fiber-optic gas sensors for carbon monoxide (CO) detection: Room temperature operating CO gas sensor using ZnO sensing film has been developed. The sensor operates based on the surface plasmon resonance (SPR) mechanism. This sensor has been reported to have a high sensitivity of 0.091${}^{\circ }$/ppm and a fast response time of about 1 s towards a wide CO concentration range of 0.5–100 ppm at room temperature. These sensors are shown to selective towards CO, with a negligible interference with other gases such as NH${}_{3}$, CO${}_{2}$, NO${}_{x}$, LPG and H${}_{2}$. Therefore, ZnO thin film-based fiber-optic sensors have been shown as potential candidates for commercial applications of CO detection [66].

### 5.2. Photonic Crystal Gas Sensors

Photonic crystal (PhC) are refractive index-based sensors that commonly employ periodic arrangements of dielectric materials with different refractive index for detection [67]. These sensors have been proposed as potential candidates to achieve high sensitivity with ability of detecting nano-meter size chemical compounds, detect environmental parameters such as temperature, pressure, and humidity, gain sensor design flexibility, provide greater security by averting electromagnetic interference from electrical signals, as well as to reduce device dimension on an integrated optical circuit platform. High-performance PhC gas sensors have been developed and reported, such as with an LOD in ppm range [68] and dissolved avidin concentration range of only 1 μm/mL [67].

#### 5.2.1. Structure and Mechanism

PhC gas sensors include dielectric materials with highly periodic micro- or nano-structures or patterns. The periodic arrangement of the employed dielectric materials can create a photonic bandgap that allows certain wavelengths of light to travel through the PhC, while other light cannot propagate. This photonic property can be designed based on the properties of the target light by using different PhC materials and patterning [69]. Mid-infrared PhCs are the common example of these devices that are used to detect CO${}_{2}$, CH${}_{4}$ or CO gases as they exhibit absorption lines in mid-infrared region. In gas-sensing application, PhC sensors are used to detect the analytes by measuring the diffraction wavelength change caused by the two factors: change in effective refractive index and change in lattice distance of periodic structure [70].

The diffraction of light in PhCs and sensing mechanism can be explained by Bragg’s law:

(4) mλ=2nd·sinθ

where *m* is diffraction order, λ diffraction wavelength of light, *n* effective refractive index, *d* the lattice distance between adjacent periodic patterns and θ incident angle. Assuming that position of the incident light and PhC layer are fixed, the effective refractive index becomes the only variable to change the allowed wavelength of the light. In most cases, the refractive index of analytes is different from that of PhC materials. This difference in refractive index brings the change to the effective refractive index, *n*, on the boundary between periodic structure and gaseous analytes. There are cases that some analytes can bring change in physical property of the photonic materials such as swelling, which can shrink or expand the PhC, therefore, change the lattice distance, *d*. Both changes in refractive index and lattice distance can affect the resultant diffraction wavelength of probe light, λ, enabling the detection of analytes [67,70].

**Figure 13:**
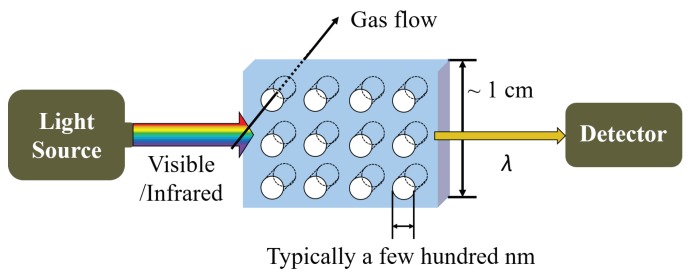
Schematic view of a photonic crystal gas sensor. Photonic crystal (PhC) with periodic micro- or nano-patterns are placed between light source and photo detector. Gaseous analytes can pass through the patterns or be condensed in the patterns where they change the effective refractive index, *n*, or the lattice distance of periodic structure, *d*. The diffraction wavelength changed by the two factors is detected by the detector based on Bragg’s law.

The typical structure of PhC gas sensors is presented in Figure 13. The PhC sensing layer is placed between the light source (ranging from infrared to visible light) and the detector [71]. Analytes are condensed onto the periodic structure or passes through the patterns. The size and the distance of each pattern are usually in sub-micro-meter range and cover 30–40% of PhC layer volume. In addition, the patterns can be optimized in various ways depending on the desired diffraction wavelength, and the size of sensing layer in PhC gas sensor can be reduced to approximately 1 cm in width [70]. The PhC gas sensors are potential candidates to further improve the conventional optical gas sensors’ properties through replacing the sensing chamber with PhCs. The conventional devices use mirrors to maintain the path of light with dimensions of ~10–50 cm therefore, they are difficult to be miniaturized. PhCs can replace this optical sensing chamber by a smaller and simpler configuration. However, the miniaturization of PhCs-based gas-sensing devices still have limitations due to the size of external light source and photo detector. PhC gas sensors can be further designed in multi-layered structure to lower LOD by 1.7 ppb. [72].

#### 5.2.2. Fabrication

PhC periodic patterns can be fabricated using advanced MEMS fabrication techniques or self-assembly methods. In MEMS fabrication techniques, advanced micro-patterning and deposition methods such as e-beam lithography, physical and CVD techniques can be employed [67,73] where the dimension and shape of the patterns can be precisely controlled. However, this can be costly compared with the simple and inexpensive self-assembly technique where micro-particles are assembled in a colloidal suspension. PhC can be synthesized by both physical and chemical method in self-assembly technique: by annealing metal thin films to produce nano-particles that can be arranged along the crystallographic orientations of substrate [74] or by drying precursor dissolved solution or colloid to induce crystallization or sedimentation in optimal condition. The methods can largely vary depending on the structure and materials used. The strong advantage of self-assembly techniques come from the simple fabrication process and low cost, especially for 3 dimensional PhC structure. However, the self-assembly can induce defects in resultant PhC structure [73].

#### 5.2.3. Applications

- Mesoporous Si-based photonic crystal gas sensors for VOC detection: Si-based photonic crystal have been researched to identify various VOCs by three differently sized patterns on one substrate. The patterns are etched on Si wafer by electrochemical anodization for mesopores with different sizes. The mesopores of 8 nm in diameter are produced in multilayer with 178, 229, and 300 nm of vertical spacings on Si substrate, which allows 430, 580, and 740 nm light to reflect. The analytes introduced onto this multi-layer-patterned PhC increases the effective refractive index and shifts the allowed wavelength of reflected light. Sensing performance of the device was tested with methanol, ethanol, and isopropanol in nitrogen carrier gas, which showed LOD in ppm range. In this method, analytes can be identified by measuring the wavelength shift gradient in time of each VOCs [68].
- Silica nano-sphere-based photonic crystal gas sensor: Self-assembled silica nano-spheres have been investigated to form photonic crystal for detection of water, ethanol and carbon disulfide ($CS_{2}$). The silica photonic crystal can be synthesized from silica colloid by drying the silica colloid on the substrate, followed by annealing at 600 ${}^{\circ }$C for sintering. This nano-structured silica can be coated with HKUST-1 to increase the interaction to analytes. The size of each silica nano-sphere is approximately 300 nm in diameter and arranged in face-centered cubic structure. Near infrared light can be introduced on [111] direction of silica nano-structure for gas-sensing test in the presence of analytes. Water, ethanol and $CS_{2}$ tested for sensing performance have showed the response time at a few seconds with the estimated detection limit at 2.6 ppm for water, 0.3 ppm for ethanol and 0.5 ppm for $CS_{2}$ [75].

## 6. Conclusions

In this review, recent progress in advanced gas sensor technology have been introduced along with the sensors’ mechanism of operation, their methods of fabrication using advanced micro- and nano-fabrication technology, the potential candidates as their sensing materials, as well as their novel applications in various field. The reviewed sensors are categorized according to their mechanism of operation to electrochemical sensors including MOS and organic-based chemiresistive sensors, CNT gas sensors, acoustic gas sensors covering QCM and SAW and capacitive micro-machined ultrasonic sensors, as well as optical sensors including fiber-optic and photonic crystal gas sensors.

Electrochemical sensors including MOS sensors and organic-based chemiresistive gas sensors offer a simple structure and may employ various conducting sensing materials. These devices operate based on measuring changes in the sensing material resistivity when exposed to target analytes. Therefore, the device property highly depends on the employed sensing materials such as metals, conducting polymer, and nano-particles. Electrochemical sensors offer several advantages for low-concentration VOC detection such as low fabrication cost and simple designs. However, their sensing performance can be easily affected by common fluctuating environmental factors; humidity and temperature. MOS sensors have long life time and short response time of sensing but have high energy consumption due to their required elevated operating temperature. Organic-based chemiresistive sensors on the other hand can operate at room temperature and have high sensitivity. However, they have poor selectivity, are temperature dependent, and are commonly affected by the relative humidity fluctuation. MOS and organic-based chemiresistive sensors’ performance can be enhanced by using porous sensing layer and nano-particles, respectively. Depending on the sensor design and employed materials, LOD in the ppb range has been reported for these sensors.

Advancement in micro- and nano-fabrication technology has led to the development of CNT sensors where they employ CNTs as their sensing material. Arc discharge, PLA, and CVD techniques are the advanced methods that can be used to fabricate CNTs. They can also be developed using various design such as conductimetric and capacitance configurations. These sensors can be divided based on their mechanism of operation to gas sorption, gas ionization, capacitive, and resonant frequency gas sensor categories. An advantage of CNT sensors arises from the flexibility of CNT functionalization with different chemical groups. However, common disadvantages of these sensors are long recovery time and their potential decreased sensitivity for low gas energy levels. CNT sensors with detection in ppb range has been reported.

Acoustic gas sensors including QCM, surface acoustic, and capacitive micro-machined ultrasonic sensors have been investigated. These sensors can detect analytes by measuring the resonance frequency change, which is a function of change in the device mass associated with the gas molecule properties and concentrations. These sensors can use various sensing materials such as polymers depending on the target gas. QCM and SAW sensors employ piezoelectric materials in their geometry. In general, piezoelectric acoustic gas sensors show very high sensitivity and fast response times. However, the sensing performance can be vulnerable to temperature changes. Piezoelectric material can be combined with micro-machining technology to increase the efficiency of these sensors. Unlike QCM and SAW, CMUTs operates based on generation of an electrostatic force between a thin suspended plate and a grounded electrode. Similar to other acoustic transducers, CMUT measures the resonance frequency shift associated with the presence of target analytes and hence, sensing material mass changes. Acoustic sensors with a level of detection in ppm to ppb range have been reported. A main reported problem associated with the acoustic sensors is their poor selectivity, which is related to their employed sensing materials.

Optical sensors including fiber-optic and photonic crystal gas sensors operates based on the detection of the light propagation through the device. Fiber-optic sensors detect analytes by measuring optical property changes of light such as wavelength, which is introduced on the polymeric sensing layer through optical fiber. These sensors have many advantages in high sensitivity, stability to environmental factors, and long lifetime. However, needs of optical fiber in the structure make it difficult to be miniaturized. Photonic crystal sensors employ periodic arrangements of dielectric materials with various refractive index. Mid-infrared PhCs are a common example of these devices to detect common gases such as CO. These sensors can be developed using advanced micro-machining technology and, therefore, the dimension and shape of the device pattern can be precisely controlled, which on the other hand increases the fabrication cost. Optical gas sensors with detection level in ppm range have been developed.
